# Genetic Diversity and Association Analysis of Traits Related to Water-Use Efficiency and Nitrogen-Use Efficiency of *Populus deltoides* Based on SSR Markers

**DOI:** 10.3390/ijms252111515

**Published:** 2024-10-26

**Authors:** Chengcheng Gao, Cun Chen, Ning Liu, Fenfen Liu, Xiaohua Su, Chenggong Liu, Qinjun Huang

**Affiliations:** 1 State Key Laboratory of Tree Genetics and Breeding, Research Institute of Forestry, Chinese Academy of Forestry, Beijing 100091, China; gaocc@caf.ac.cn (C.G.); chencun0610@163.com (C.C.); ning.liu@ugent.be (N.L.); lfflff0122@163.com (F.L.); suxh@caf.ac.cn (X.S.); 2 Key Laboratory of Tree Breeding and Cultivation, State Forestry and Grassland Administration, Beijing 100091, China; 3 UGent-Woodlab (Laboratory of Wood Technology), Department of Environment, Ghent University, 9000 Ghent, Belgium

**Keywords:** poplar, water-use efficiency, nitrogen-use efficiency, genetic variation, trait-marker association

## Abstract

*Populus deltoides* is one of the primary tree species for bioenergy production in temperate regions. In arid/semi-arid northern China, the scarcity of water and nitrogen significantly limits the productivity of poplar plantations. The identification of relevant molecular markers can promote the breeding of resource-efficient varieties. In this study, 188 genotypes of *P. deltoides* from six provenances served as experimental material. Genetic differentiation analysis, analysis of molecular variance (AMOVA), principal coordinate analysis (PCoA), unweighted pair group method with arithmetic mean (UPGMA) clustering, and genetic structure analysis were performed using selected simple sequence repeat (SSR) markers. Based on these analyses, the association analysis of water-use efficiency (WUE) and nitrogen-use efficiency (NUE) were conducted using general linear model (GLM) and mixed linear model (MLM) approaches. The results showed that 15 pairs of SSR primers successfully amplified across all 188 individuals, with an average of 7.33 alleles (Na) observed per primer pair. The polymorphism information content (PIC) ranged from 0.060 to 0.897, with an average of 0.544, indicating high genetic diversity in the selected markers. The average inbreeding coefficient intra-population (Fis), inbreeding coefficient inter-population (Fit), and inter-population genetic fraction coefficient (Fst) values were 0.005, 0.135, and 0.132, respectively, indicating high heterozygosity, substantial inbreeding within populations, and moderate genetic differentiation, with an average gene flow (Nm) of 1.964, suggesting substantial gene flow between populations. Additionally, molecular variance was primarily within individuals (84.12%). Genetic structure analysis revealed four subgroups, with some degree of genetic admixture among the provenances. In the GLM model, 11 markers were significantly associated with five traits (*p* < 0.05), with an average contribution rate of 15.82%. Notably, SSR132 and SSR143 were significantly associated with multiple traits (*p* < 0.05). The MLM model identified two markers (SSR47 and SSR85) significantly associated with ground diameter (*p* < 0.05) and one marker (SSR80) significantly associated with NUE (*p* < 0.05). This study identifies loci associated with WUE and NUE, laying a foundation for future genetic improvement and marker-assisted breeding strategies in poplar.

## 1. Introduction

Woody plants are a vital resource for providing abundant lignocellulosic biomass. With the increasing global demand for renewable energy and sustainable materials, it is projected that, by 2030, woody plants could contribute up to 300 million tons of dry biomass [1,2]. Among these, poplar (*Populus*) stands out due to its rapid growth, leading to its widespread cultivation [3,4]. China possesses the largest area of poplar plantations globally, covering approximately 8.5 million hectares, accounting for one-third of the world’s total poplar plantation area [5]. The collection, preservation, and evaluation of existing poplar germplasm resources, along with the selection and breeding of important target traits, are fundamental prerequisites for advancing the development of poplar resources, enhancing productivity, and ensuring a sustainable supply of wood.

Since the 1950s, large-scale poplar plantations have been established in northern China. Over half a century of development has led to the selection and widespread cultivation of poplar varieties with superior growth performance. However, these plantations are primarily located in arid/semi-arid northern China with poor soil quality, where the scarcity of water and nitrogen has become the primary abiotic limitations to sustainable productivity [3,6]. In addition, the continuous growth of stands, along with the implementation of extensive operational and management methods, has resulted in the water consumption of most poplar plantations surpassing local precipitation levels [7]. This scenario, the deterioration of soil fertility due to frequent logging [8,9], has increasingly become a critical pressure that restricts the growth and productivity of these plantations. Previous research has demonstrated that breeding and cultivating poplar varieties with high water-use efficiency (WUE) and nitrogen-use efficiency (NUE) can significantly alleviate this pressure [10,11]. Therefore, there is an urgent need in arid/semi-arid northern China to develop new poplar varieties that efficiently utilize water and nitrogen to establish fast-growing, high-yielding plantations.

In the 20th century, traditional breeding methods significantly improved poplar productivity by utilizing genetic resources from gene pools, such as regional varieties, wild relatives, and introduced species, to select superior varieties [12]. For example, two *P*. *deltoides* clones, I-69 (*P. deltoides* Bartr. ‘Lux’) and I-63 (*P. deltoides* Bartr. ‘Harvord’), were introduced from Italy to China in 1972 and widely adopted due to their excellent fast-growing characteristics [13]. However, with the rapid development of the modern timber industry, greenhouse effects, and the frequent occurrence of extreme drought events, traditional hybrid breeding has struggled to meet the industry’s evolving demands due to its long breeding cycles and high resource requirements [14]. On the other hand, traditional research methods for evaluating water and nutrient utilization often face limitations due to their destructive nature or restrictive conditions [11]. Studies have shown that the abundance of stable carbon isotopes (δ^13^C) is a reliable indicator of long-term internal WUE in C_3_ plants, and its abundance is mainly related to internal leaf concentration and atmospheric CO_2_ (C_i_/C_a_) ratio [15,16,17]. The abundance of stable nitrogen isotopes (δ^15^N) can be utilized to detect and quantify nitrogen inputs and losses and is used to characterize plant NUE [18,19]. Unfortunately, most poplar species are outcrossing plants, and their WUE and NUE are controlled by multiple quantitative trait loci (QTLs), with complex genetics [20]. Based on these limitations, we have a limited understanding of the genetic basis of WUE and NUE in poplar, further hindering the development of new varieties with efficient water and nitrogen use. Therefore, utilizing modern molecular biology techniques to study the genetic basis of WUE and NUE in poplar and accelerating the breeding process is an urgent research priority.

Marker-assisted selection (MAS) has significantly shortened breeding cycles and improved breeding efficiency [21]. Based on this, the study by Chen et al. [22] showed that the combination of gene expression and association mapping could help identify candidate genes underlying the QTL of interest and complement map-based cloning and MAS, ultimately enhancing breeding progress and improving WUE in cereal varieties (e.g., wheat). Additionally, the discovery of low N-tolerant genotypes may help in the identification of the genomic regions responsible for NUE and its deployment in pearl millet breeding programs through MAS [23]. It is well known that the key to MAS is identifying the molecular markers associated with target traits, but the current lack of effective markers related to these traits has impeded progress in the genetic selection and breeding of poplar. Association analysis, which is based on linkage disequilibrium between allelic variations at different loci, is an effective method for identifying the markers associated with quantitative traits, especially when there is abundant natural genetic variation [24,25,26].

With advancements in molecular biology, various types of molecular marker have been developed, such as amplified fragment length polymorphism (AFLP) [27], restriction fragment length polymorphism (RFLP) [28], simple sequence repeat (SSR) [29], and single nucleotide polymorphism (SNP) [30]. Among these, SSR markers are considered ideal for association analysis due to their co-dominance, multi-allelic nature, stability, wide genome coverage, and ease of detection [31,32]. In recent years, researchers have successfully developed SSR markers through data mining in different *Populus* species (*P. tomentosa* [18], *P. simonii* [33], and *P. deltoides* [34]) and applied them to studies of quantitative traits, such as growth characteristics [24], wood properties [25], and physiological traits [35,36]. However, due to limited population sizes, complex trait genetics, and technical hurdles, studies on SSR markers associated with WUE and NUE remain scarce. Moreover, poplar, is a model species for important target trait studies in woody plants [37]. Importantly, poplar has many advantages (e.g., rapid growth, ease of reproduction, a small genome, significant genetic diversity, and abundant genetic resources), and these attributes make it an ideal reference species for MAS in forest trees [38]. Therefore, studying poplar for MAS is anticipated to enhance our understanding of resource utilization in woody plants and enhancing our ability to address issues such as long breeding cycles, slow results, and low phenotypic selection accuracy in tree breeding.

*P. deltoides* is a key tree species in temperate regions, widely distributed across North America, and is one of the primary perennial woody species used in bioenergy production. *P. deltoides* is dioecious, with high genetic diversity and heterozygosity due to widespread pollen and seed dispersal [39]. This genetic diversity makes it an ideal parental species for poplar hybrid breeding, widely used in tree breeding programs to produce high-yielding commercial varieties for pulp, paper, and bioenergy industries [40,41]. While *P. deltoides* exhibits high productivity, it also has high water and nitrogen demands [10]. In the arid/semi-arid areas of northern China, high WUE and NUE are crucial for the successful propagation and regional planting of *P. deltoides*. However, most studies to date have focused on the breeding of fast-growing varieties [42], the physiological characteristics [43], and the molecular regulatory mechanisms of growth and development [44], with extremely limited research on the genetic basis of WUE and NUE, especially in terms of their genetic variation, population structure, and QTL mapping. Even though, in 2016, Fahrenkrog et al. [34] first reported on association studies of bioenergy traits in *P. deltoides*, research on their resource-use efficiency is still lacking. This lack of research restricts the selection and promotion of resource-use efficient breeding materials. Therefore, in this study we analyzed 188 *P. deltoides* individuals collected from 22 sites across six provenances. Under uniform water and nitrogen conditions, we focused on the performance of different genotypes of *P. deltoides* in terms of growth rate (height and ground diameter), carbon storage (total biomass), and resource-use efficiency (WUE and NUE). Our primary objectives were to (1) evaluate the phenotypic and molecular genetic diversity of the *P. deltoides* population, (2) elucidate the population structure of *P. deltoides*, and (3) identify SSR markers associated with traits related to WUE and NUE of *P. deltoides*.

## 2. Results

### 2.1. Performance and Correlation of WUE and NUE

**Figure 1:**
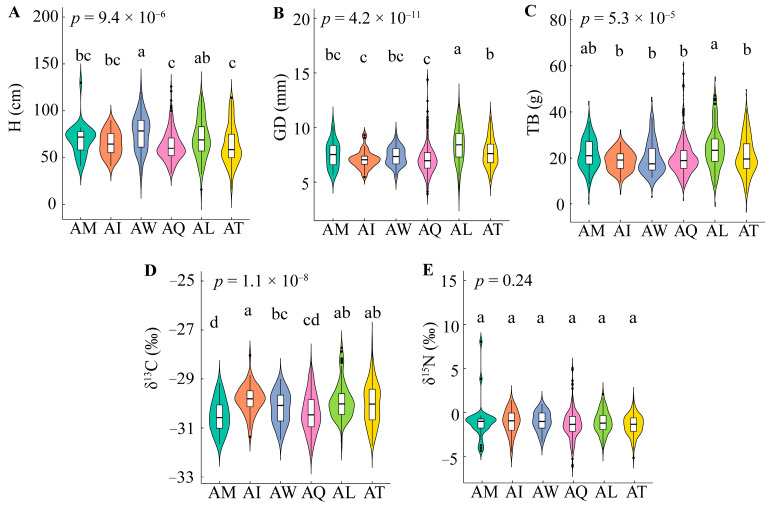
Variation in WUE and NUE among 6 provenances. Panels (**A**–**E**) show violin plots for H (**A**), GD (**B**), TB (**C**), δ^13^C (**D**), and δ^15^N (**E**). *p* value is the significant value of difference under ANOVA. The different letters indicate a significant difference (*p* < 0.05), as determined by Duncan’s multiple range test. Provenances: AM: Missouri, USA; AI: Iowa, USA; AW: Washington, USA; AQ: Quebec, Canada; AL: Louisiana, USA; AT: Tennessee, USA. H: Height; GD: Ground diameter; TB: Total biomass; δ13C: Carbon stable isotope; δ15N: Stable isotope of nitrogen.

**Figure 2:**
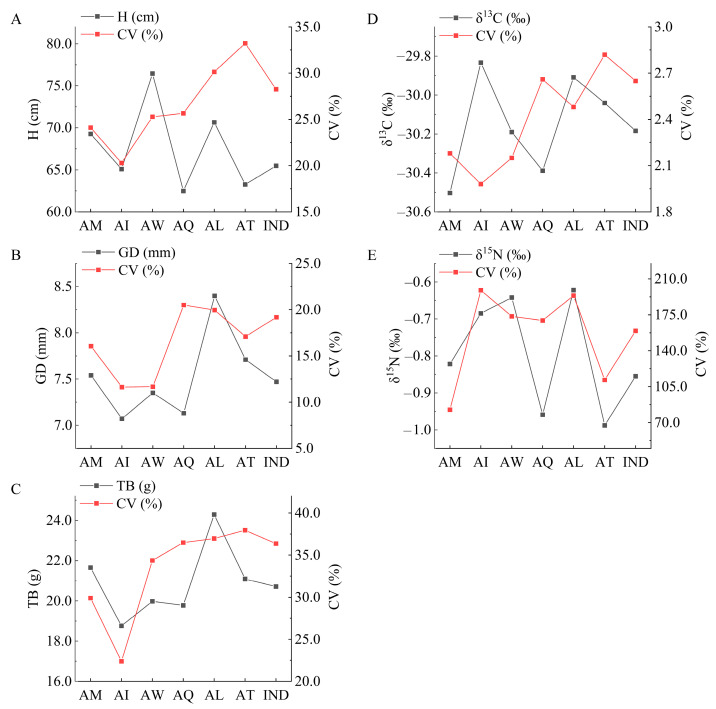
Mean values and coefficient of variation (CV) regarding traits of different provenances: H (**A**), GD (**B**), TB (**C**), δ^13^C (**D**), and δ^15^N (**E**). The horizontal coordinates are the names of the different groups. The double ordinates are the mean value of traits (left) and the CV of corresponding trait (right). Provenances: AM: Missouri, USA; AI: Iowa, USA; AW: Washington, USA; AQ: Quebec, Canada; AL: Louisiana, USA; AT: Tennessee, USA. IND: 188 individuals. H: Height; GD: Ground diameter; TB: Total biomass; δ^13^C: Carbon stable isotope; δ^15^N: Stable isotope of nitrogen.

**Figure 3:**
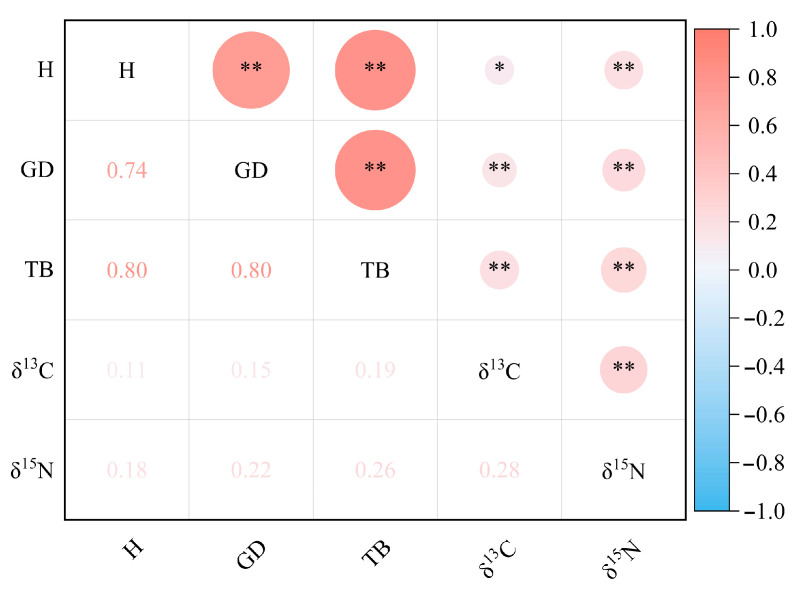
Correlation coefficient between different traits. * indicates *p* < 0.05 and ** indicates *p* < 0.01. The right color column represents the correlation coefficient. H: Height; GD: Ground diameter; TB: Total biomass; δ^13^C: Carbon stable isotope; δ^15^N: Stable isotope of nitrogen.

Figure 1 shows that all traits except δ^15^N showed significant differences (*p* < 0.01) among provenances. Each provenance exhibited distinct superior traits: the AW had significantly higher seeding height (H), the AL had greater ground diameter (GD) and total biomass (TB), and the AI exhibited higher δ^13^C compared to other provenances. However, the δ^13^C of AI was significantly different from that of AM, AW, and AQ (*p* < 0.05), but not from that of AL and AT (*p* > 0.05). At the provenance level, the variation ranges for H, GD, TB, δ^13^C, and δ^15^N were 62.49 to 76.45 cm, 7.07 to 8.40 mm, 18.76 to 24.29 g, −30.50‰ to −29.833‰, and −0.98‰ to 0.622‰, respectively. At the individual level, the mean values for H, GD, TB, δ^13^C, and δ^15^N were 65.47 cm, 7.47 mm, 20.72 g, −30.184‰, and −0.855‰, respectively (Figure 2). The coefficients of variation (CV) for different provenances ranged from 20.28% to 33.23% for H, 11.62% to 20.50% for GD, 22.38% to 37.95% for TB, 1.98% to 2.82% for δ^13^C, and 82.59% to 198.89% for δ^15^N. At the individual level, the CVs were 28.25% for H, 19.19% for GD, 36.36% for TB, 2.65% for δ^13^C, and 159.41% for δ^15^N. The traits showed varying degrees of variation, with the following order at both the provenance and individual levels: δ^15^N > TB > H > GD > δ^13^C (Figure 2). Figure 3 indicates that all traits were significantly positively correlated (*p* < 0.01), except for H and δ^13^C, which showed a significant positive correlation (*p* < 0.05).

### 2.2. Genetic Differentiation and Genetic Diversity Analysis of P. deltoides Populations

In Table 1, 15 pairs of SSR primers detected a total of 110 alleles (Na) for all 188 individuals, with the number of alleles per primer pair ranging from 2 (SSR85) to 18 (SSR120 and SSR126), averaging 7.33. The effective number of alleles (Ne) ranged from 1.07 (SSR85) to 10.49 (SSR120), with an average of 3.62. SSR120 and SSR126 were highly informative, with information index (I) values of 2.502 and 2.603, respectively. The average observed heterozygosity (Ho) and expected heterozygosity (He) were 0.883 and 0.905 for Ho and 0.755 and 0.903 for He. Most loci (except SSR85 and SSR104) showed heterozygosity deficiency (He > Ho). The polymorphism information content (PIC) ranged from 0.062 (SSR85) to 0.905 (SSR120), with an average value of 0.585. The mean inbreeding coefficients Fis and Fit were 0.005 and 0.135, respectively, while the fixation index, Fst, ranged from 0.054 to 0.201, with an average of 0.132. Furthermore, the gene flow (Nm) among provenances was relatively high, with an average of 1.964. Across all SSR markers, 13.33% of the heterozygotes exhibited significant excess, and 12 markers showed significant deviations from Hardy–Weinberg equilibrium (HWE) (*p* < 0.01).

As shown in Table 2, the AW exhibited relatively low genetic diversity (Na = 2.67, Ne = 1.92, I = 0.609, Ho = 0.371, He = 0.344). The AQ, AL, and AT had higher genetic diversity, with I values of 1.133, 1.130, and 1.160, respectively. Additionally, these three populations all possessed private alleles. Table 3 shows that 84.12% of the genetic variation was attributed to differences of individuals, 11.85% to differences intra-provenances, and 4.03% to differences between individuals. Chi-square tests showed that genetic distance was significantly correlated with latitude (χ^2^ = 1143.242, *p* < 0.05).

### 2.3. Population Structure and Phylogenetic Relationships

**Figure 4:**
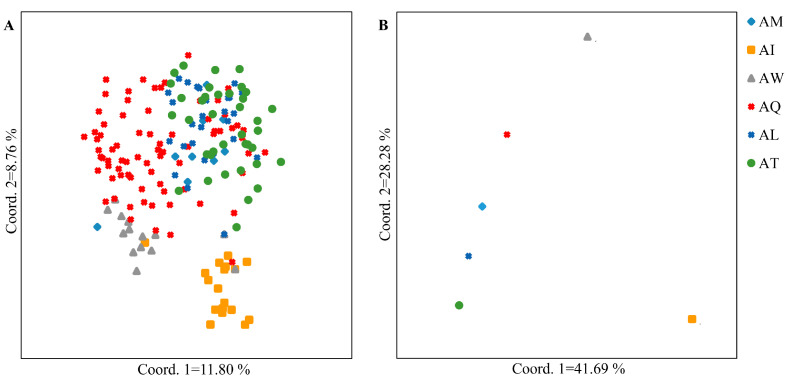
Principal component analysis (PCoA) of 188 individuals (**A**) and 6 provenances (**B**).

**Figure 5:**
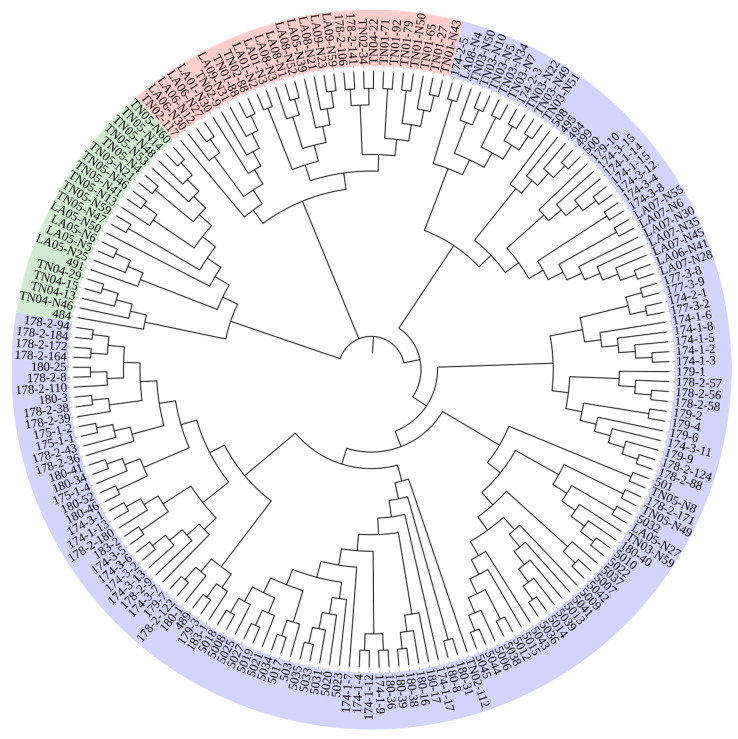
Cluster analysis (UPGMA) of 188 individuals of *P. deltoides*. Samples are colored based on the subpopulation assignments made by PowerMarker.

Both principal coordinate analysis (PCoA) and unweighted pair group method with arithmetic mean (UPGMA) clustering divided the *P. deltoides* individuals into three groups. The PCoA results (Figure 4A) indicate that the first group mainly comprises individuals from the AW; the second group includes individuals primarily from the AM, AQ, AL, and AT; and the third group consists mainly of individuals from the AI. The UPGMA clustering results (Figure 5) similarly show that the first and second groups mainly consist of individuals from the AL and AT, respectively, while the third group is a mixture of individuals from all six provenances.

To further understand the genetic structure of the six *P. deltoides* populations, we conducted PCoA analysis on the provenances (Figure 4B). The results demonstrate that the AM, AQ, AL, and AT cluster together, while the AW and AI are genetically distinct from the other populations.

**Figure 6:**
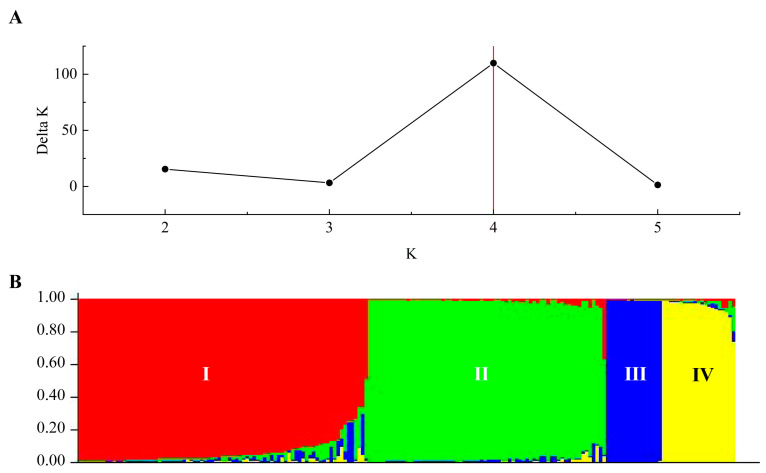
Changes in ΔK values at different K values (**A**) and the population genetic structure at K = 4 (**B**). K represents the number of subgroups in (**A**). Samples in (**B**) are colored according to the subpopulation they were assigned to by Structure.

Bayesian clustering analysis (Figure 6A) revealed that when K = 4, the ΔK value is relatively large, indicating that the 188 *P. deltoides* individuals can be divided into four distinct groups. The genetic structure analysis (Figure 6B) shows that most individuals from AM, AT, and AL belong to the red group (Group I), most individuals from AI and AQ belong to the green group (Group II), individuals from AW are primarily assigned to the blue group (Group III), and a subset of AQ individuals belongs to the yellow group (Group IV).

### 2.4. SSR Linkage Disequilibrium Analysis and Trait-Marker Association

**Figure 7:**
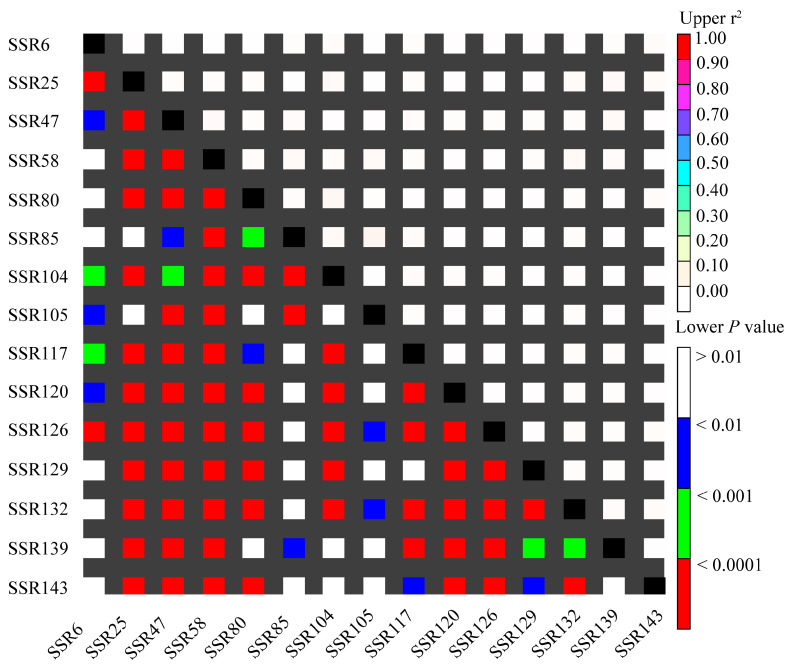
The distribution of LD among 15 SSR markers. r^2^: Linkage disequilibrium coefficient; *P*: Observed haplotype frequency.

Figure 7 shows the combinations of loci in linkage. Among the 105 pairwise SSR locus combinations, linkage disequilibrium (LD) was observed in both collinear and non-collinear combinations (non-white grids below the diagonal). Among the 105 pairs of markers (r^2^ range of 0.001–0.059), statistical significance (*p* < 0.01) supported LD in 78 pairs of SSR loci, accounting for 74.29% of all pairwise combinations. According to r^2^, only 0.01% (r^2^ ≥ 0.05) of the paired loci exhibited significant LD, with most loci being in linkage equilibrium (r^2^ < 0.05; *p* < 0.01).

Using the general linear model (GLM) and mixed linear model (MLM) modules in TASSEL 2.1, association analysis was performed for each trait (Table 4). The GLM identified 11 SSR loci significantly associated with five important traits. Among these, two loci were associated with H, two loci with TB, three loci with GD, three loci with δ^13^C, and five loci with δ^15^N. In this analysis, two loci were found to be significantly associated with multiple traits: SSR132 was significantly associated with H, TB, and GD, with phenotypic contribution rates of 13.09%, 12.62%, and 11.77%, respectively; SSR143 was significantly associated with TB and H, with phenotypic contribution rates of 7.10% and 6.81%, respectively. The MLM identified three SSR loci significantly associated with two important traits (Table 5): SSR47 and SSR85 were associated with GD and SSR80 was associated with δ^15^N.

## 3. Discussion

In arid/semi-arid regions, poplar cultivation still faces the challenge of high input and low output, primarily due to low WUE and NUE. Currently, hybrid progenies of *P. deltoides* × *P. nigra* or *P. deltoides* × *P. cathayana* are considered better adapted to these areas [45], with notable varieties including *P*. × *euramericana* ‘74/76’ [46] and *P.* × *canadensis* ‘Zhongliao 1’ [7] in northern China. Introduced *P. deltoides* is becoming one of the main parent materials for future poplar breeding in China [13,40]. Therefore, studying the genetic basis of WUE and NUE will help address the contradiction between the demand for superior poplar varieties and the insufficient supply of parent materials, thereby fundamentally promoting the efficient and sustainable development of poplar breeding.

Accurate assessment and description of phenotypic trait variation among different plant genotypes is crucial for the successful selection of resource-efficient varieties/clones [47]. In this study, we observed significant differences in H, GD, and TB of different *P. deltoides* species, as well as notable variations in WUE (δ^13^C), aligning with the findings of Yin et al. [48]. This result further substantiates the notion that long-term natural selection contributes to increased WUE [11]. Furthermore, a higher coefficient of variation indicates greater diversity in phenotypic traits and enhanced environmental adaptability [49]. In our study, the coefficients of variation for WUE and nitrogen-use efficiency (NUE)-related traits were notably high (>10%), suggesting a diversification of individual genetic information in *P. deltoides* and a substantial selection potential, consistent with previous research findings [8,11].

As is known to all, early assessment of germplasm genetic diversity can help prevent production losses due to genetic vulnerability [50]. Additionally, understanding the genetic diversity within populations is crucial for using molecular marker techniques to study tree population evolution and perform association analysis [51]. At the molecular level, genetic diversity refers to the degree of gene variation within a population. The number and types of alleles controlling different phenotypes of the same trait on homologous chromosomes directly reflect the genetic diversity of the population [52]. Currently, the use of SSR markers to study genetic diversity in poplars is quite mature [40,53,54]. In this study, 15 SSR primer pairs detected a total of 110 alleles, with an average of 7.33 alleles per primer pair, exceeding the alleles per primer pair of *P. tomentosa* [53] and *P. wulianensis* [54], indicating a rich genetic diversity in different sources of *P. deltoides*.

Furthermore, PIC values reflect the polymorphism level of the primers, typically categorized as low (<0.25), medium (0.25 < PIC < 0.5), or high (>0.5) [55,56]. In this study, the average PIC value of the 15 primer pairs indicated moderate polymorphism, higher than that of *P. tomentosa* [53] and *Ficus carica* [57], suggesting that these primers are suitable for analyzing genetic diversity in *P. deltoides* populations. The study also showed that the level of genetic differentiation between or among populations can be categorized based on the differentiation coefficient (Fst) into low (<0.05), moderate (0.05 < Fst < 0.15), high (0.15 < Fst < 0.25), and very high (>0.25) [58]. In this study, the average Fst value was 0.132, indicating moderate genetic differentiation, with 86.8% of the variation occurring within populations, much lower than the genetic differentiation levels observed in *P. davidiana* and *P. tremula* [59]. This suggests that, in the selection of *P. deltoides*, more emphasis should be placed on selecting individuals within populations.

Additionally, gene flow refers to the exchange of genetic material between different populations [60]. Mushtaq et al. [61] suggested that gene flow could be caused by the migration of individuals or gametes (e.g., pollen) between populations, or mating between individuals from different populations. We found a high Nm value (>1), suggesting substantial gene flow before introduction, providing abundant material for hybrid breeding selection. This high level of genetic diversity is consistent with the findings of Fahrenkrog et al. [34], meeting the requirements for subsequent trait-association analysis. However, the high gene flow within the population may lead to increased genetic homogeneity among closely related populations, serving as a warning signal for the potential erosion of genetic diversity in the future [50,62]. Therefore, it is crucial to establish core breeding populations as soon as possible to effectively preserve superior alleles.

In reality, heterozygous defects or excesses often cause deviations in population gene frequencies and genotype frequencies from HWE [63]. This study revealed that, among all SSR markers, 13.33% of heterozygotes exhibited significant excess and 12 markers demonstrated significant deviation from HWE. These findings are consistent with previous research on *P. tremula* [64]. The observed deviations may be attributed to factors such as inbreeding, non-random mating, or disruption of population structure [65], which further suggests the potential existence of population substructure within *P. deltoides* population. Therefore, analyzing the population structure not only reflects gene exchange and affinity among individuals but also serves as a prerequisite for association mapping, which helps improve mapping efficiency and avoid false positives [66]. In this study, population structure analysis was performed at both individual and provenance levels. While there were some differences among the results from PCoA, UPGMA, and Bayesian clustering, it is noteworthy that AQ clustered with others. In the structure of *P. tomentosa*, similar combinations of different provenances were observed, primarily due to genetic exchange resulting from similar elevation patterns across different geographic origins [67]. Geographically, AQ may have dispersed along the St. Lawrence River–Ohio River–Mississippi River corridor, leading to gene flow with different provenances, especially with those located upstream of the Mississippi River, such as AT, AL, and AM. This indicates that AQ has a broad genetic background and high adaptability to various environments, further supporting the feasibility of early selection of *P. deltoides* provenance and genotypes. Moreover, we found that individuals or provenances distributed in the Mississippi River basin clustered together, showing a certain degree of genetic similarity, which is consistent with previous research findings [34]. Unlike the two subpopulations reported previously [40], this study, using Bayesian clustering, divided the *P. deltoides* association population into four subpopulations. The genetic information suggests a certain degree of genetic mixing among provenances, which we speculate is due to differences in population size and the number of individuals within populations [26]. Therefore, future studies on population structure should not ignore the impact of population size and should focus on utilizing the AQ gene pool to further explore excellent alleles for efficient water use.

LD analysis informs both the genetic basis for association mapping and the choice of appropriate strategies [68]. Studies show that the feasibility of trait-association depends on low LD levels [69], as seen in species like *P. tomentosa* [70,71], *Eucalyptus* [72], *Gossypium hirsutum* [73], and *Paeonia rockii* [26]. Our research also observed low LD levels between SSRs, primarily due to *P. deltoides* being a cross-pollinated species with high recombination rates. This indicates a complex and diverse provenance population, which generates a wide range of genetic variation through gene exchange and recombination, potentially allowing the identification of genes responsible for key trait variations through association genetics. Additionally, significant human interventions, such as hybridization [74], controlled pollination [75], and germplasm exchange [76], can also cause varying degrees of changes in LD levels, which should not be overlooked and warrant consideration by poplar breeders worldwide.

Overall, the application of association analysis in molecular marker-assisted breeding effectively accelerates the selection of superior varieties [77,78], and this is no exception in model species like poplar. Research indicates that differences in root responses to heterogeneous nutrients affect growth, development, and relative competitive ability, thereby impacting productivity [79]. To minimize the effects of environmental heterogeneity, we assessed the association between traits and markers under the same water and nitrogen conditions. The GLM method identified 11 SSR markers associated with five traits. The K + Q combined MLM further reduced the effective SSR markers to three (associated with two traits) by eliminating false positives, thereby enhancing the accuracy of the association results. Furthermore, traits were found to be not only significantly associated with SSRs but also exhibited pleiotropy or co-localization, as previously reported [26,71,80]. We hypothesize that these pleiotropic associations help identify important genomic regions and are valuable for trait improvement using marker-assisted selection (MAS). However, differences in population size and structure can lead to variations in association results. A typical association population should consist of multiple independent and unrelated individuals from the same region [81], a condition successfully met in studies of soybean [82], maize [83], and wheat [84]. Therefore, to reduce false positives and provide accurate estimates of allele variation, future research should validate association results using verification populations from different regions or molecular biology experiments.

## 4. Materials and Methods

### 4.1. Test Materials

**Figure 8:**
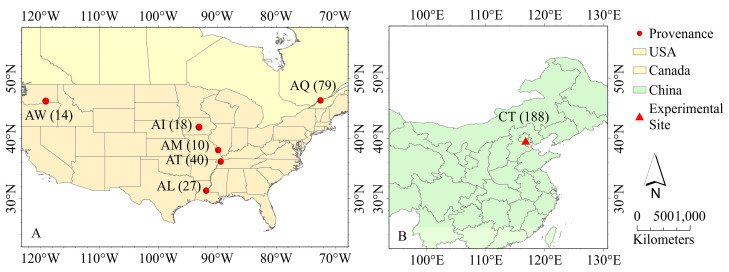
Distribution map of *P. deltoides* provenances and test sites. (**A**) shows the geographical locations of the six provenances of *P. deltoides* in the United States and Canada, with the number of samples collected for each provenance in brackets. (**B**) shows the geographical location of the test site in Tongzhou, Beijing, China, with the number of test materials in brackets. The legend, compass, and scale are on the right. Provenances: AM: Missouri, USA; AI: Iowa, USA; AW: Washington, USA; AQ: Quebec, Canada; AL: Louisiana, USA; AT: Tennessee, USA. Experimental site: CT: Beijing Tongzhou, China.

From 2009 to 2014, our research team collected the germplasm resources in the main distribution areas of *P. deltoides*, including six provenances (Missouri (AM), Iowa (AI), Washington (AW), Tennessee (AT), Louisiana (AL), and Quebec (AQ)), with a total 188 genotypes (Figure 8A). In April 2016, 1-year-old cuttings (15 cm in length, 1.5 cm in diameter) of each genotype were rooted and cultured in plastic pots (25 cm in height, 18 cm in caliber) filled with substrate: one plant per pot, 12 pots per genotype. The substrate volume ratio was loess:charcoal:coarse sand = 6:1:1. The pH value of the substrate was 6.61, the volume moisture content is about 45.43%, and the maximum moisture content is about 63.88%. The plants were then cultivation for 120 days in a greenhouse at the Tongzhou Experimental Nursery of the Chinese Academy of Forestry (116°45′06″ E, 39°44′01″ N) (Figure 8B, Table S1). During the seedling cultivation period, the greenhouse temperature was maintained at 24–26 °C, with relative air humidity ranging from 40% to 80%. In August, 9 plants from each genotype were selected and transferred, along with the pots, to an open field of the experimental base outside the greenhouse for further cultivation. The field cultivation adopted a completely randomized block design, including three blocks; each block had 188 genotypes and three plants per genotype. Watering was 1200 mL every two days, and manual weeding and insect removal were performed every half a month.

### 4.2. Growth and Physiological Trait Determination

In early September 2016, three plants of each genotype were selected, and 3–5 mature functional leaves were collected from each plant. The leaves were placed at 105 °C for 2 h, then dried at 75 °C for 36 h. After the samples were cooled, they were ground through a 100-mesh sieve for isotope determination. The ^13^C to ^12^C ratio and the ^15^N to ^14^N ratio in the samples were determined using a DELTA V Advantage isotope ratio mass spectrometer (Thermo Fisher Scientific, Inc., Waltham MA, USA). The isotope determination was mainly assisted by Beijing Helesi Biological Co., Ltd. (Beijing, China).

In October 2016, seedling height and ground diameter were measured. After the measurement, the whole plant (including leaves, roots, and stems) was harvested and placed in a 75 °C oven to constant weight. Then, the total biomass was measured. Each growth trait of each genotype was repeated 3 times, and the average value was finally taken.

### 4.3. DNA Extraction and Primer Amplification

Leaves were collected during the growing season and stored in a −40 °C refrigerator. The total genomic DNA from the leaves was extracted using the highly efficient modified cetyltrimethylammonium bromide (CTAB) method [85]. The quality and integrity of the extracted genomic DNA were assessed by 1% agarose gel electrophoresis. The DNA concentration was then determined using a NanoDrop-2000 ultramicro spectrophotometer (Thermo Fisher Scientific, Waltham, MA, USA). Finally, the DNA was diluted to 50 ng·μL^−1^ and stored at −20 °C for polymerase chain reaction (PCR) amplification.

The SSR primers utilized in this study were derived from our team’s previous research findings [40]. Specifically, 145 pairs of poplar SSR primers were randomly selected from the DNA of 20 unrelated individuals. Among these, 75 pairs were developed based on the functional gene sequences of poplar [33,53,86], while 70 pairs were sourced from the International Populus Genome Consortium (IPGC, http://www.ornl.gov/sci/ipgc/ssr_resource.htm, accessed on 10 March 2019) and Washington University (Poplar Molecular Genetics Cooperative, http://poplar2.cfr.washington.edu, accessed on 12 March 2019). The stability and polymorphism of these SSR primers was assessed through PCR amplification, 2% agarose gel electrophoresis, and 8% nondenaturing polyacrylamide gel electrophoresis. Following preliminary screening and detection, a total of 15 pairs of polymorphic SSR primers were selected after excluding null alleles at the locus. These primers were ultimately employed to analyze the genetic diversity and population structure of 188 *P. deltoides* individuals.

### 4.4. Data Processing

Genetic diversity parameters were calculated using GeneAlEx (Version 6.503, Australian National University, AUS, Canberra, Australia), including the number of alleles (Na), effective number of alleles (Ne), Shannon’s information index (I), observed heterozygosity (Ho), expected heterozygosity (He), number of private alleles, gene flow (Nm), F-statistics (Fis: inbreeding coefficient intra-population; Fit: inbreeding coefficient inter-population; Fst: in-ter-population genetic fraction coefficient), and polymorphism information content (PIC) [87]. GeneAlEx was also used for Hardy–Weinberg equilibrium (HWE) testing, analysis of molecular variance (AMOVA), and principal coordinate analysis (PCoA). Cluster analysis based on the unweighted pair-group method with arithmetic mean (UPGMA) was performed using PowerMarker (Version 3.25, North Carolina State University, Raleigh, NC, USA), and the results were visualized using the iTOL (Version v6, EMBL-EBI, Heidelberg, Germany) online tool (https://itol.embl.de/).

Population genetic structure was analyzed using Structure (Version 2.3.4, Pritchard Lab, Stanford University, Stanford, CA, USA), which employs a model-based clustering algorithm within a Bayesian framework and Markov chain Monte Carlo (MCMC) methods. To determine the optimal number of subpopulations (K), each K value from 1 to 7 was run independently 10 times. Each run consisted of a 100,000-step burn-in period followed by 100,000 MCMC iterations. The optimal K was estimated using the ΔK parameter, based on the rate of change in the log probability of data between consecutive K values, according to the model developed by Evanno et al. [88]. Individuals with a membership probability (Q) of 0.75 or higher were assigned to their respective clusters, while those with a Q value of less than 0.75 were assigned to the admixture group. Finally, kinship coefficients were calculated using the SPAGeDi (Version 1.3, Free Univ Brussels, Brussels, Belgium) software package [89].

In TASSEL (Version 2.3.4, Buckler Lab, Cornell University, Ithaca, NY, USA), two models were used to assess the effects of population structure (Q) and relatedness (K) on marker-trait associations for five traits: (i) a simple GLM model, akin to a standard analysis of variance without considering Q and K, and (ii) an MLM model that simultaneously accounts for Q and K [90,91]. Trait and molecular data were organized using Excel (Version 2016, Microsoft Corp, Albuquerque, NM, USA), plotted using R (Version 4.2.2, R Foundation for Statistical Computing, Vienna, Austria). One-way ANOVA and Duncan’s methods were employed for ANOVA and multiple comparisons (α = 0.05) using SPSS (Version 21.0, IBM Corp, Armonk, NY, USA). Additionally, the Chi-square test (χ^2^) and Pearson correlation coefficient (r) were utilized to analyze the correlation between variables (α = 0.05) using the same SPSS (Version 21.0, IBM Corp, Armonk, NY, USA).

## 5. Conclusions

*P. deltoides* is one of the key germplasm materials for future poplar breeding in China. Identifying molecular markers significantly associated with its WUE and NUE is an effective approach to accelerate efficient breeding research. In this study, significant differences were observed among the 188 *P. deltoides* germplasm resources in traits such as seedling height, ground diameter, biomass, WUE, and NUE, highlighting the high genetic diversity and strong environmental adaptability. Trait variation intra-provenances is a major driver of this diversity, indicating that future breeding efforts should prioritize genotype identification and selection. Population structure analysis revealed that *P. deltoides* can be divided into four subpopulations, with gene flow among sources being a significant contributor to the rich genetic diversity. Additionally, special attention should be given to the exploitation of superior alleles within the AQ source. Notably, this study successfully identified three SSR molecular markers significantly associated with ground diameter and NUE: SSR47 and SSR85 were associated with ground diameter, while SSR80 was associated with NUE. These markers can facilitate the rapid screening of high-nitrogen fertilized plants and the identification of genes associated with efficient water and nitrogen utilization. However, to obtain reliable results from association analyses in actual research, it is essential to consider not only the representativeness of the research population (i.e., multiple independent and unrelated individuals from the same area) but also the genetic diversity and molecular biological characteristics of various target traits (e.g., stress resistance, rapid growth, and efficient resource utilization). Therefore, based on the findings of this study, it will be crucial to continue phenotypic identification of multiple traits across various locations and to enhance and validate the correlation analysis results through genomics and other methodologies. The approaches will be vital for improving the breeding efficiency and resource conservation of poplar and other woody plants.

## Figures and Tables

**Table 1:** The genetic diversity parameters of 15 SSR primers for 188 *P. deltoides* individuals. Na: number of alleles per locus; Ne: effective number of alleles; I: Shannon’s information index; Ho: observed heterozygosity; He: expected heterozygosity; PIC: polymorphic information content; HWE: Hardy–Weinberg equilibrium; * indicates significant deviations from HWE (* *p* < 0.05, ** *p* < 0.01, *** *p* < 0.001); Fis: inbreeding coefficient intra-population; Fit: inbreeding coefficient inter-population; Fst: inter-population genetic fraction coefficient; Nm: gene flow.

Locus	Na	Ne	I	Ho	He	PIC	HWE (*p*)	Fis	Fit	Fst	Nm
SSR6	4	2.43	1.108	0.543	0.589	0.546	0.024 *	0.000	0.065	0.065	3.567
SSR25	8	3.56	1.530	0.601	0.719	0.686	0.000 ***	−0.004	0.244	0.247	0.763
SSR47	7	2.22	0.963	0.452	0.550	0.464	0.002 **	0.176	0.290	0.139	1.553
SSR58	6	2.61	1.251	0.473	0.617	0.578	0.000 ***	0.006	0.205	0.201	0.995
SSR80	7	2.15	1.059	0.505	0.534	0.488	0.002 **	−0.010	0.105	0.114	1.935
SSR85	2	1.07	0.141	0.064	0.062	0.060	0.651	−0.078	−0.020	0.054	4.390
SSR104	4	1.73	0.639	0.447	0.421	0.337	0.960	−0.140	−0.013	0.112	1.981
SSR105	9	1.53	0.809	0.309	0.347	0.332	0.000 ***	0.019	0.101	0.084	2.744
SSR117	7	4.71	1.654	0.713	0.788	0.756	0.016 *	−0.031	0.134	0.160	1.313
SSR120	18	10.49	2.502	0.883	0.905	0.897	0.000 ***	−0.100	0.010	0.100	2.250
SSR126	18	10.33	2.603	0.755	0.903	0.896	0.000 ***	−0.036	0.074	0.106	2.108
SSR129	9	4.35	1.751	0.691	0.770	0.742	0.000 ***	0.049	0.189	0.147	1.449
SSR132	4	3.65	1.339	0.527	0.726	0.677	0.000 ***	−0.044	0.149	0.185	1.103
SSR139	3	2.00	0.741	0.441	0.500	0.388	0.325	−0.070	0.060	0.121	1.817
SSR143	4	1.53	0.647	0.234	0.347	0.316	0.000 ***	0.340	0.435	0.144	1.484
Total	110	54.36	--	--	--	--	--	--	--	--	--
Mean	7.33	3.62	1.249	0.509	0.585	0.544	--	0.005	0.135	0.132	1.964

**Table 2:** The genetic diversity parameters of different *P. deltoides* provenances. Na: number of alleles per locus; Ne: effective number of alleles; I: Shannon’s information index; Ho: observed heterozygosity; He: expected heterozygosity. Provenances: AM: Missouri, USA; AI: Iowa, USA; AW: Washington, USA; AQ: Quebec, Canada; AL: Louisiana, USA; AT: Tennessee, USA.

Provenance	Na	Ne	I	Ho	He	Number of Private Alleles
AM	3.73	2.65	0.908	0.453	0.484	--
AI	2.93	1.91	0.644	0.463	0.371	--
AW	2.67	1.92	0.609	0.371	0.344	--
AQ	6.13	3.05	1.133	0.505	0.551	18
AL	5.00	3.36	1.130	0.583	0.562	3
AT	5.33	2.99	1.160	0.550	0.589	4
Mean	4.30	2.65	0.931	0.488	0.484	--

**Table 3:** Analysis of molecular variance (AMOVA) of 188 *P. deltoides* individuals. df: degree freedom; SS: Stdev square; MS: Mean square; Est. Var: Estimated variance.

Source	df	SS	MS	Est. Var.	Percentage Variation (%)
Among Provenances	5	170.584	34.117	0.538	11.85
Among Individuals	182	761.693	4.185	0.183	4.03
Within Individuals	188	718.000	3.819	3.819	84.12
Total	375	1650.277		4.540	100.00

**Table 4:** Contribution rates of associated markers with traits in GLM models.

Trait	Locus	df	*F*-Value	*p*-Value	Contribution Rate (%)
H	SSR132	9	3.051	0.002	13.09
TB	SSR132	9	2.918	0.003	12.62
GD	SSR132	9	2.848	0.004	11.77
GD	SSR47	8	2.906	0.005	10.75
δ^13^C	SSR58	15	1.991	0.019	13.89
δ^15^N	SSR126	69	1.551	0.019	48.15
δ^13^C	SSR129	27	1.723	0.022	21.13
δ^15^N	SSR132	9	2.221	0.023	10.26
δ^15^N	SSR80	13	1.967	0.026	13.02
δ^15^N	SSR104	4	2.764	0.029	5.79
δ^15^N	SSR120	65	1.492	0.031	44.87
TB	SSR143	6	2.346	0.033	7.10
δ^13^C	SSR117	20	1.713	0.036	15.98
H	SSR143	6	2.248	0.041	6.81
GD	SSR85	1	4.092	0.045	2.02
Mean					15.82

**Table 5:** Estimate vale of alleles of associated markers with traits in MLM models.

Trait	Locus	df	*F*-Value	*p*-Value	lnLikelihood
GD	SSR47	8	2.6707	0.009	−2.70 × 10^2^
	SSR85	1	4.7035	0.031	−2.86 × 10^2^
δ^15^N	SSR80	12	2.1396	0.017	−2.71 × 10^2^

## Data Availability

The data underlying this article are available in the article and in its Supplementary Materials.

## Supplementary Materials

The following supporting information can be downloaded at: https://www.mdpi.com/article/10.3390/ijms252111515/s1.
