# Supplementary material for: Genetic Diversity and Association Analysis of Traits Related to Water-Use Efficiency and Nitrogen-Use Efficiency of Populus deltoides Based on SSR Markers
Source: Int J Mol Sci. 2024 Oct 26;25(21):11515. doi: 10.3390/ijms252111515 (PMC11547121; doi:10.3390/ijms252111515)
Supplement: Supplementary file 1 [file ijms-25-11515-s001.zip › ijms-3215153-supplementary.pdf]

Supplementary Materials:

Table S1 Test genotypes and provenances. Provenances: AM: Missouri, USA; AI: Iowa, USA; AW: Washington, USA; AQ: Quebec, Canada; AL: Louisiana, USA; AT: Tennessee, USA.

Number	Provenance_Number	Genotype	Number	Provenance_Number	Genotype
1	AM_1	484	95	AQ_53	178-2-9
2	AM_2	489	96	AQ_54	178-2-94
3	AM_3	491	97	AQ_55	179-1
4	AM_4	494	98	AQ_56	179-10
5	AM_5	495	99	AQ_57	179-2
6	AM_6	499	100	AQ_58	179-3
7	AM_7	500	101	AQ_59	179-4
8	AM_8	501	102	AQ_60	179-6
9	AM_9	503	103	AQ_61	179-7
10	AM_10	508	104	AQ_62	179-9
11	AI_1	5006	105	AQ_63	180-1
12	AI_2	5007	106	AQ_64	180-16
13	AI_3	5008	107	AQ_65	180-17
14	AI_4	5009	108	AQ_66	180-25
15	AI_5	5010	109	AQ_67	180-3
16	AI_6	5012	110	AQ_68	180-31
17	AI_7	5013	111	AQ_69	180-34
18	AI_8	5014	112	AQ_70	180-36
19	AI_9	5015	113	AQ_71	180-38
20	AI_10	5036	114	AQ_72	180-39
21	AI_11	5037	115	AQ_73	180-40
22	AI_12	5038	116	AQ_74	180-41

23	AI_13	5039	117	AQ_75	180-46
24	AI_14	5041	118	AQ_76	180-52
25	AI_15	5042	119	AQ_77	180-8
26	AI_16	5043	120	AQ_78	183-1
27	AI_17	5044	121	AQ_79	183-2
28	AI_18	5045	122	AL_1	LA01-N2
29	AW_1	5017	123	AL_2	LA01-N3
30	AW_2	5018	124	AL_3	LA01-N30
31	AW_3	5019	125	AL_4	LA05-N25
32	AW_4	5020	126	AL_5	LA05-N27
33	AW_5	5021	127	AL_6	LA05-N39
34	AW_6	5022	128	AL_7	LA05-N5
35	AW_7	5023	129	AL_8	LA05-N50
36	AW_8	5025	130	AL_9	LA05-N6
37	AW_9	5027	131	AL_10	LA06-N12
38	AW_10	5031	132	AL_11	LA06-N27
39	AW_11	5032	133	AL_12	LA06-N30
40	AW_12	5033	134	AL_13	LA06-N41
41	AW_13	5034	135	AL_14	LA07-N28
42	AW_14	5035	136	AL_15	LA07-N30
43	AQ_1	174-1-12	137	AL_16	LA07-N35
44	AQ_2	174-1-13	138	AL_17	LA07-N45
45	AQ_3	174-1-14	139	AL_18	LA07-N55
46	AQ_4	174-1-15	140	AL_19	LA07-N6
47	AQ_5	174-1-17	141	AL_20	LA08-N1
48	AQ_6	174-1-2	142	AL_21	LA08-N21

49	AQ_7	174-1-3	143	AL_22	LA08-N3
50	AQ_8	174-1-4	144	AL_23	LA08-N4
51	AQ_9	174-1-5	145	AL_24	LA08-N52
52	AQ_10	174-1-6	146	AL_25	LA09-N23
53	AQ_11	174-1-7	147	AL_26	LA09-N31
54	AQ_12	174-1-8	148	AL_27	LA09-N59
55	AQ_13	174-1-9	149	AT_1	TN01-27
56	AQ_14	174-2-1	150	AT_2	TN01-65
57	AQ_15	174-2-3	151	AT_3	TN01-71
58	AQ_16	174-2-5	152	AT_4	TN01-79
59	AQ_17	174-3-1	153	AT_5	TN01-88
60	AQ_18	174-3-11	154	AT_6	TN01-92
61	AQ_19	174-3-12	155	AT_7	TN01-N43
62	AQ_20	174-3-13	156	AT_8	TN01-N50
63	AQ_21	174-3-15	157	AT_9	TN02-112
64	AQ_22	174-3-2	158	AT_10	TN02-117
65	AQ_23	174-3-4	159	AT_11	TN02-34
66	AQ_24	174-3-5	160	AT_12	TN02-88
67	AQ_25	174-3-6	161	AT_13	TN02-9
68	AQ_26	174-3-7	162	AT_14	TN03-N10
69	AQ_27	174-3-8	163	AT_15	TN03-N22
70	AQ_28	175-1-1	164	AT_16	TN03-N34
71	AQ_29	175-1-2	165	AT_17	TN03-N49
72	AQ_30	175-1-4	166	AT_18	TN03-N5
73	AQ_31	177-3-2	167	AT_19	TN03-N50
74	AQ_32	177-3-8	168	AT_20	TN03-N51

75	AQ_33	177-3-9	169	AT_21	TN03-N59
76	AQ_34	178-2-106	170	AT_22	TN03-N7
77	AQ_35	178-2-110	171	AT_23	TN04-13
78	AQ_36	178-2-122	172	AT_24	TN04-15
79	AQ_37	178-2-124	173	AT_25	TN04-22
80	AQ_38	178-2-141	174	AT_26	TN04-29
81	AQ_39	178-2-164	175	AT_27	TN04-N46
82	AQ_40	178-2-171	176	AT_28	TN05-N13
83	AQ_41	178-2-172	177	AT_29	TN05-N14
84	AQ_42	178-2-180	178	AT_30	TN05-N21
85	AQ_43	178-2-184	179	AT_31	TN05-N4
86	AQ_44	178-2-36	180	AT_32	TN05-N41
87	AQ_45	178-2-38	181	AT_33	TN05-N46
88	AQ_46	178-2-39	182	AT_34	TN05-N47
89	AQ_47	178-2-43	183	AT_35	TN05-N48
90	AQ_48	178-2-56	184	AT_36	TN05-N49
91	AQ_49	178-2-57	185	AT_37	TN05-N53
92	AQ_50	178-2-58	186	AT_38	TN05-N59
93	AQ_51	178-2-8	187	AT_39	TN05-N60
94	AQ_52	178-2-88	188	AT_40	TN05-N8
